# “Transcriptomics”: molecular diagnosis of inborn errors of metabolism via RNA-sequencing

**DOI:** 10.1007/s10545-017-0133-4

**Published:** 2018-01-25

**Authors:** Laura S. Kremer, Saskia B. Wortmann, Holger Prokisch

**Affiliations:** 1 Institute of Human Genetics, Technische Universität München, Trogerstrasse 32, 81675 Munich, Germany; 2 Institute of Human Genetics, Helmholtz Zentrum München, Munich, Germany; 3 Department of Pediatrics, Paracelsus Medical University Salzburg, Salzburg, Austria

## Abstract

Exome wide sequencing techniques have revolutionized molecular diagnostics in patients with suspected inborn errors of metabolism or neuromuscular disorders. However, the diagnostic yield of 25–60% still leaves a large fraction of individuals without a diagnosis. This indicates a causative role for non-exonic regulatory variants not covered by whole exome sequencing. Here we review how systematic RNA-sequencing analysis (RNA-seq, “transcriptomics”) lead to a molecular diagnosis in 10–35% of patients in whom whole exome sequencing failed to do so. Importantly, RNA-sequencing based discoveries cannot only guide molecular diagnosis but might also unravel therapeutic intervention points such as antisense oligonucleotide treatment for splicing defects as recently reported for spinal muscular atrophy.

## Introduction

The ascension of next generation sequencing (NGS) techniques has revolutionized molecular diagnostics. Especially whole exome sequencing (WES) has subsequently become the first tier approach for clinical diagnostics of Mendelian disorders, e.g., mitochondrial disorders or other inborn errors of metabolism (IEM) (Wortmann et al 2017).

Despite its tremendous impact, the diagnostic yield of WES analysis is far from complete. For mitochondrial disorders, a diagnosis is achieved in about 50% of the cases (Taylor et al 2014; Wortmann et al 2015; Pronicka et al 2016), whereas in other disease groups even more patients remain genetically undiagnosed (O’Donnell-Luria and Miller 2016).

**Fig. 1:**
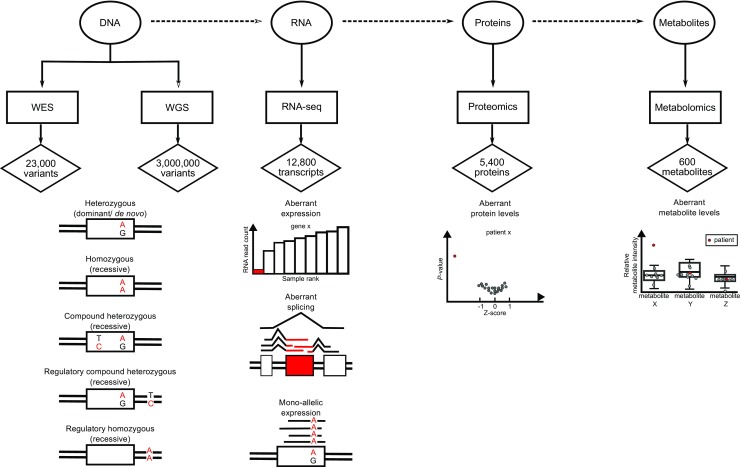
Multiomics discoveries. Genetic information, stored in the form of the biopolymer DNA, is exploited to produce messenger and operative biopolymers like RNA and proteins. Active biopolymers frequently produce intermediate or final biochemical moieties as metabolites. Methods for the investigations of these species are depicted in boxes and the numbers provided in diamonds represent typical results (for whole exome sequencing = WES and whole genome sequencing = WGS performed on blood or fibroblasts, RNA-seq and proteomics performed on fibroblasts, and metabolomics performed on plasma). The lower panel shows representative observations for the respective method Figure adapted from (Kremer, unpublished doctoral thesis, Technische Universität München))

To understand this unsatisfactory outcome, one first needs to recapitulate that disorders not following a Mendelian (autosomal dominant/recessive, X-linked dominant/recessive) or mitochondrial inheritance, like imprinting disorders, polygenetics, or repeat expansions, cannot easily be detected using WES (Fig. 1). Furthermore it is important to realize that WES is the exclusive sequencing of the protein coding (exonic) regions of the genome compromising only about 2% of the 3*10^9^ human genomic nucleotides (Bamshad et al 2011; Rabbani et al 2014; Petersen et al 2017). As 85% of the annotated Mendelian disease-causing variants reside in the exonic regions, this restricted analysis, however, allows cost-effective sequencing in large scale (van Dijk et al 2014; Petersen et al 2017). Vice versa, it implies that a significant proportion of pathogenic variants residing outside the exonic regions are invisible to WES. The difficult analysis and interpretation of these non-coding variants has likely resulted in an underestimation of their contribution to disease.

Due to decreasing sequencing costs it will soon be feasible to perform whole genome sequencing (WGS) instead of WES, allowing almost complete coverage of the genome. While WGS enables the detection of most variants, whether coding or non-coding, equally tremendous advances in characterizing these non-coding variants have not yet been met (Soemedi et al 2017). An average WES analysis yields between 20,000–23,000 variants per individual (Bamshad et al 2011; Wieland 2015). To identify the pathogenic variant(s) among them, comprehensive filtering and prediction of variant effects are necessary. For IEM, mostly autosomal recessively inherited, filtering for allele frequency of less than 1% and for genes harboring bi-allelic variants is frequently employed, narrowing down the list to rare potentially deleterious bi-allelic variants in about 5–25 genes. Further prioritization of these variants relies on functional evidence, e.g., that they lie in previously disease-associated genes or in genes encoding proteins known to be important for a given pathway, organelle, or phenotype (Haack et al 2010). Frequently, the functional implication of a variant cannot be predicted with sufficient reliability referred to as variant of unknown significance (VUS). WGS, in turn, reveals about 3 to 5 million variants per individual rendering prioritization even more challenging. Considering pathogenic non-coding variants likely distorting gene expression, we here give an overview of how genome-wide detection of expression perturbation by RNA-sequencing (RNA-seq) analysis adds crucial functional evidence to the genetic information obtained by WES and WGS and enables an increase in the diagnostic yield for IEM.

## Specifications and implications of RNA-seq

The discovery of reverse transcriptases in 1970 and their potential to generate cDNA from RNA in 1971 enabled the adaptation of existing methods for DNA analysis to concordantly investigate RNA species (Baltimore 1970; Temin and Mizutani 1970; Spiegelman et al 1971). Subsequently, in 1990, the reverse transcriptase polymerase chain reaction (RT-PCR) was developed to amplify RNA followed by appropriate platforms allowing RNA sequencing (Shaffer et al 1990). As for DNA, such sequencing endeavors were initially limited to single gene analysis while the development of NGS in 2005 eventually enabled global transcriptome analysis (Margulies et al 2005). Nowadays, Illumina provides the most widely used sequencing platform (Goodwin et al 2016). The commonly employed Illumina TruSeq RNA Library preparation protocol is optimized for an input of 0.1 to 1 μg of total high quality RNA (RNA integrity number (RIN) > 8) derived from various human tissues (TruSeq® RNA Sample Preparation v2 Guide (Illumina)). However, they also provide solutions for low quality samples with reduced RNA yield (10–20 ng RNA from fresh or frozen tissue). The library is prepared by first purifying mRNAs by poly(A) selection. Subsequently, the RNA is fragmented and reverse transcribed into cDNA. The cDNA molecules are then further processed by end repair and A-tailing to facilitate adapter ligation and library enrichment by PCR. Following quality control and quantification, libraries from different samples can be pooled in equimolar amounts and finally be subjected to sequencing. Additional protocols have been established for the investigation of other types of RNA like micro RNA.

The thereby gained unobscured view on the cellular transcriptome provides comprehensive information on mRNA quality and quantity as well as possible perturbations thereof. It not only permits the detection of genetic variants at the mRNA sequence level but allows direct probing of the effect of genetic variants by assessing, e.g., altered expression levels, aberrant splicing, or gene fusions (Byron et al 2016).

While the first clinical implications of RNA analysis were relatively simple and mainly focussed on analysis for the presence of the expression of certain viral genes to detect infections with RNA viruses, the applicability and complexity of transcriptome analysis steadily increased (Ito et al 1999; Byron et al 2016).

It is nowadays widely used as a prognostic outcome measure, e.g., by assessing the expression of certain gene sets aiding treatment decisions for breast cancer or leukemia, and for monitoring immune responses hinting at possible rejections following organ transplantation (Byron et al 2016). In particular, within the cancer field, the diagnostic power of RNA-seq has subsequently become evident where it is readily used to detect gene fusions. Gene fusions are the result of chromosomal rearrangements which are frequently encountered in cancer cells (Maher et al 2009; Stephens et al 2009). While such rearrangements are also detectable by conventional DNA sequencing techniques, not all such translocations result in the expression of fusion genes and hence may not have any functional consequences (Levin et al 2009). RNA-seq, in turn, allows direct detection of the pathogenic expression of fusion genes and is now routinely used as a diagnostic tool in cancer research (Mitelman et al 2007; Ozsolak and Milos 2011).

However, for Mendelian disorders, RNA expression analysis was so far not employed as a diagnostic tool and focused on single gene or single case investigations. A commonly encountered expression aberration, though, is aberrant splicing (Tazi et al 2009; Singh and Cooper 2012; Scotti and Swanson 2015) (Fig. 1). Aberrant splicing can be caused by splice site mutations, mutations in splicing factor-binding sites, as well as by intronic variants. Mutations in canonical splice sites are usually recognized in WES or WGS data. However, in only a few conditions the predicted effect has been validated at the RNA level, as there is no biomaterial available or assay established. For the remainder, pathogenicity is not established and the variant therefore remains a VUS.

**Fig. 2:**
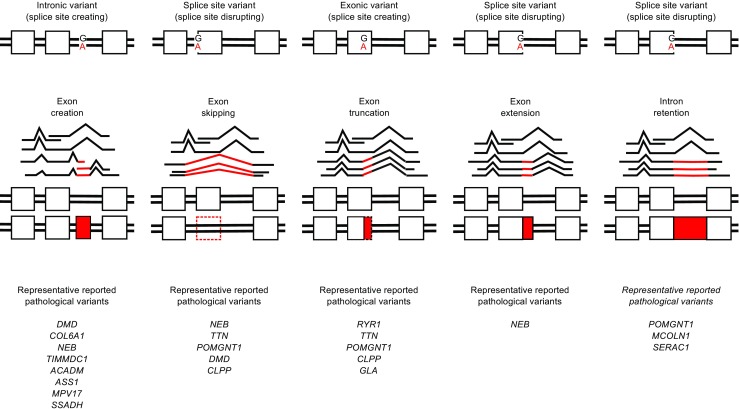
Splicing aberrations. Coding and non-coding variants depicted in the upper panel can provoke distinct splicing aberrations shown in the middle panel. Examples for each instance are listed in the lower panel

Variants affecting splicing can provoke 1) exon skipping as reported for various IEM (e.g., for *ACADM* (Medium Chain Acyl CoA dehydrogenase deficiency) (Korman et al 2004), *ASS1* (Citrullinemia) (Kimani et al 2015), *MPV17* (mitochondrial DNA depletion syndrome) (Navarro-Sastre et al 2008), and *SSADH* (Succinic semialdehyde dehydrogenase deficiency) (Chambliss et al 1998); 2) exon skipping and creation of a novel exon; 3) intron retention as seen in *SERAC1* (MEGDEL syndrome, (Wortmann et al 2012; Morel et al 2006); and 4) exon truncations of various forms as seen in *GLA* (Fabry disease) (Chang et al 2017) (Fig. 2).

Another evolving theme regarding transcriptional perturbation in Mendelian disorders is mono-allelic expression (MAE, Fig. 1) where only one allele is expressed, whereas the other allele is transcriptionally silenced or post-transcriptionally degraded based on genetic and epigenetic grounds. Epigenetic mechanisms provoking MAE broadly fall into three categories: random MAE of autosomal genes; random MAE of X-chromosomal genes; and exclusive expression from either the paternal or maternal allele due to parent-of-origin imprinting (as reviewed by (Fahrner and Bjornsson 2014)). Genetic perturbations can include mutations affecting splicing or generating premature stop codons and subsequently provoking nonsense mediated mRNA decay (NMD) of the affected allele, alterations in promoter or regulatory regions, as well as large deletions. Therefore, MAE detected by RNA sequencing can provide crucial evidence for the implication of variants either not detected with WES (e.g., variants in introns or regulatory regions) or not prioritized after WGS (e.g., VUS). Heterozygous coding variants compound with these unrecognized variants resulting in allele silencing can then mimic the effects of homozygous variants at the RNA level. This is especially important when investigating recessive disorders as the compound heterozygous variants might not have been prioritized otherwise.

## Systematic RNA-seq analysis for Mendelian disorders

Until recently, it was unclear how many rare and strong RNA alterations are present in an individual because it was not systematically investigated. This information is, however, crucial when implementing RNA-seq as a diagnostic tool. To tackle this question, two recent studies performed large scale RNA-seq on patient material. Cummings et al performed RNA-seq on patient derived muscle in a cohort of 50 patients with rare genetically unsolved muscle disorders (Cummings et al 2017). They first characterized aberrant splicing in patients previously diagnosed with splice site mutations. Subsequently this information was used to develop an algorithm that could detect aberrant exon-exon junctions which were present only in patient material but not in reference muscle RNA-seq data generated in the Genotype-Tissue Expression (GTEx) Consortium project (Consortium 2015). The latest GTEx release (dbGaP Accession phs000424.v7.p2) provides RNA-seq data for 53 tissues from 714 donors and depicts the most comprehensive reference dataset. Authorized access to the data is possible upon request. To identify rare (by Cummings et al referred to as unique) splicing events they only considered outlier events that were maximal in a given sample and less than half in the next highest sample. This analysis revealed unique splicing events in 190 genes per individual.

A similar rationale was applied by us in a study comprising 105 fibroblast cell lines from suspected mitochondrial disease patients (Kremer et al 2017). We adapted an algorithm for splicing quantitative trait loci (QTLs) to a rare disease setting where we detected novel splice sites by comparing one sample against all others. We could identify five aberrant splicing events per sample using a stringent cut-off of a Hochberg adjusted *P*-value smaller than 0.05. To additionally determine genes whose expression was outside their physical range, we computed expression outliers using a combination of Z-score, which is simply a measure of fold change considering the inter-sample variance, and statistical testing. As before, we adapted the filters for a rare disease setting and determined rare and strong events with a Hochberg adjusted *P*-value smaller than 0.05 and a Z-score larger than 3. This resulted in the median detection of one expression outlier per sample. Finally, we also investigated the median MAE genes per sample. We filtered for heterozygous rare (minor allele frequency (MAF) < 0.001) single nucleotide variants (SNVs) with an RNA-seq coverage of more than 10 reads and considered the SNVs mono-allelically expressed when more than 80% of the reads harbored that variant and the Hochberg adjusted *P*-value was smaller than 0.05. This revealed a median of six MAE events per sample. Altogether, the number of strong and rare RNA defects per sample is rather small (median = 12) and, therefore, allows manual inspection to clarify a pathogenic impact of the aberration.

Consequently, in both studies, the pipelines were applied to patients for whom WES or WGS was not conclusive, with the aim to investigate the usability of RNA-seq as a diagnostic tool. Indeed, the authors could identify pathogenic variation by computing aberrant expression, MAE, and aberrant splicing. Both studies revealed aberrant splicing as the most frequently observed pathogenic aberration. The detected aberrant splicing events comprised exon skipping, exon truncation, exon creation, and intron retention and were caused by coding as well as non-coding variants.

Notably, most of the exonic variants were VUS that previously evaded variant prioritization. RNA-seq analysis provided crucial functional evidence for their pathological relevance. Even more strikingly, aberrant splicing was also evoked by putatively synonymous variants. Cummings et al report a synonymous variant in *RYR1* causing an exonic splice gain as the novel splice site is stronger than the canonical splice site. In *POMGNT1* they showed exon skipping as a result of a synonymous variant disrupting a splice motif. Importantly, detection of aberrant splicing did not only provide crucial information on exonic variants but also enabled the identification of pathogenic non-coding variants. Cummings et al found a hemizygous intronic variant in *DMD* resulting in the creation of a novel exon yielding a premature stop codon in three patients. In four patients, an intronic variant in *COL6A1* exerted the same effect of pseudoexon creation. Interestingly, they identified this variant in 27 additional patients where it occurred independently.

Similarly, we detected a homozygous deep intronic variant in *TIMMDC1* in three families also creating a pseudoexon and resulting in a premature stop codon. *TIMMDC1* also appeared as an expression outlier in investigated samples, most probably due to nonsense mediated RNA decay (NMD) as result of the premature stop codon.

Taken together, both studies clearly demonstrate the power of RNA-seq to reliably detect pathogenic RNA defects that were not obvious solely from genetic information. Cummings et al achieved a diagnostic yield of 35% (by solving 17/50 patients), while our study reached a diagnostic rate of 10% (5/48 patients were solved) (Table 1). It is noteworthy that the two studies differ in their approach due to the characteristic of the investigated patient cohort and expected genetic defects where both scenarios are likely encountered in a clinical setting. Cummings et al performed their study in a phenotypically stratified patient cohort. Patients with overlapping clinical signs and symptoms potentially share the same genetic cause resulting in a common RNA defect among these patients. For the detection of rare RNA effects, it is therefore extremely important to employ large control datasets, for example GTEx.

**Table 1:** Diagnostic yield of RNA-sequencing

Disorder	Average diagnostic yield WES/WGS	Diagnostic yield before RNA-sequencing	Diagnostic yield after RNA-sequencing
Rare muscular disorders (Cummings et al 2017)	25–50% (Yang et al 2014; Ankala et al 2015; Chong et al 2015; Taylor et al 2015)	0/50 = 0% (0 solved, 4 VUS, 12 candidate genes, 34 no candidates after WES/WGS)	17/50 = 35% (17 solved, 2 VUS, 8 candidate genes, 27 no candidates)
Mitochondrial disorders (Kremer et al 2017)	39–60% (Taylor et al 2014; Wortmann et al 2015; Pronicka et al 2016)	0/48 = 0%	5/48 = 10%

*VUS* variant of unknown significance, *WES* whole exome sequencing, *WGS* whole genome sequencing

In contrast, we investigated suspected mitochondrial disease patients presenting with diverse clinical phenotypes. Therefore, since the patients were likely not affected by mutations in the same gene, hence resulting in diverging RNA defects, the samples served as good controls for each other. Furthermore, Cummings et al restricted their analysis to known disease associated-genes and, therefore, employed less stringent filtering while we screened genome-wide requiring more stringent filtering criteria. In the first case the pipeline is more sensitive for small changes, which is feasible when focusing on a limited set of genes. The second genome-wide study is less sensitive and only focuses on strong outliers, but is able to detect a cryptic exon in a gene that was previously not known to be associated with a mitochondrial disorder *(TIMMDC1).*

Importantly, our study revealed aberrant splicing in two patients at a position to which background splicing was evident in controls, consistent with a previous report that cryptic splice sites are often not entirely repressed but active at low levels (Kapustin et al 2011). Our systematic analysis confirmed that 70% of the private exons arose from weak splice sites. These weak splicing events are usually dismissed as ‘noise’ since they are only supported by a few reads in a given sample. Our analysis showed that they could be detected as accumulation points across multiple individuals: weakly spliced cryptic exons are loci more susceptible to turn into strongly spliced sites than other intronic regions. This observation may help in the future to detect variants causing cryptic splicing.

## Limitations of RNA-seq (“the tissue is the issue”)

As outlined in both studies, tissue specific expression is a major obstacle in transcriptome analysis. So far, there is no systematic study whether disease causing variants cluster in genes which are tissue specifically or ubiquitously expressed. Cummings et al report a poor expression of genes commonly affected in muscle disease in blood and fibroblasts. On the other hand, in fibroblasts, we detected expression of 2574 of the 3768 disease genes (68%) listed in OMIM. Therefore, even though the affected tissue is not available, the RNA-seq analysis of an unaffected tissue can still provide crucial evidence for molecular diagnostics. To the contrary, performing the analysis in unaffected tissue might benefit the detection of primary perturbations, as regulatory secondary consequences on other genes are restricted (Li et al 2017).

Secondary effects that are common in the investigated samples will be removed by appropriate filtering for rare effects. In contrast, specific secondary effects for a given mutation can only be distinguished from the primary defect upon careful dissection of the underlying molecular variation.

Furthermore, detection of splicing aberrations requires sophisticated bioinformatic tools as splicing can be extremely complex. Alternative splicing is seen for 94% of genes (Ward and Cooper 2010), while different isoforms might be expressed in different tissues or even coexist in a given tissue. The alterations can come in many flavors, where exons can be shortened, removed, or created and introns can be retained. Existing prediction tools largely facilitate reduction of the complexity and limit the number of the detected events. However, as is true for all prediction tools, these tools are not perfect and the predicted events need further experimental validation. Discoveries from RNA-seq analysis are largely limited to variants causing an RNA defect. Generally, RNA-seq does not provide any functional evidence easing the prioritization of missense mutations.

## Multiomics

With increasing numbers of quantitative metabolomic studies and readily available platforms, quantitative metabolomics, specifically for IEM, will sooner or later complement molecular DNA and RNA analysis in diagnostic settings. The small number of established gene-metabolite associations limit the genome-wide endeavors to interrogate metabolomics data to prioritize pathogenic variants. For established associations integration of metabolomics data has already proven fruitful for variant prioritization (Abela et al 2016; van Karnebeek et al 2016).

## Concluding remarks

Initial studies have shown the successful application of RNA-seq analysis to complement inconclusive WES and WGS studies for mitochondrial disease and neuromuscular disorders (additional yield of 10–35%). Future endeavors increasing sample sizes will allow even more sophisticated statistical analysis and will further enable the detection of moderate events. To achieve the maximal outcome of such investigations we have to join forces on a global level. Especially in the field of rare disorders, the benefits of sharing data among global registries to increase sample size and statistical power are evident. As for sharing WES and WGS, ethical concerns need to be addressed and instances for data protection need to be in place.

Finally, with increasing success of therapeutic modulation of aberrant splicing, as recently shown for spinal muscular atrophy (Finkel et al 2017), and in preclinical studies also for IEM (Matos et al 2014; Coelho et al 2015; Lee et al 2016; Chang et al 2017), further treatment strategies for disorders discovered by RNA-seq might be developed.
